# 3D bioprinting and its potential impact on cardiac failure treatment: An industry perspective

**DOI:** 10.1063/1.5128371

**Published:** 2020-02-18

**Authors:** Ravi K. Birla, Stuart K. Williams

**Affiliations:** 1 BIOLIFE4D, Houston, Texas 77021, USA; 2 Bioficial Organs Program, University of Louisville, Louisville, Kentucky 40202, USA

## Abstract

3D printing technologies are emerging as a disruptive innovation for the treatment of patients in cardiac failure. The ability to create custom devices, at the point of care, will affect both the diagnosis and treatment of cardiac diseases. The introduction of bioinks containing cells and biomaterials and the development of new computer assisted design and computer assisted manufacturing systems have ushered in a new technology known as 3D bioprinting. Small scale 3D bioprinting has successfully created cardiac tissue microphysiological systems. 3D bioprinting provides an opportunity to evaluate the assembly of specific parts of the heart and most notably heart valves. With the continuous development of instrumentation and bioinks and a complete understanding of cardiac tissue development, it is proposed that 3D bioprinting may permit the assembly of a heart described as a total biofabricated heart.

## INTRODUCTION

Heart failure is a major medical problem globally and most times requires a heart transplantation. However, the number of donor organs available for transplant is always significantly lower than the number of patients who require a heart transplant. The patients, who do receive a heart transplant, require life-long immune suppression therapy, which significantly hinders the quality of life. There are currently more than 6.2 million patients in the US with heart failure, and heart failure accounted for 78 356 mortalities in 2016.^1^ There is a very large economic cost associated with heart failure, reported to be $30.7 billion in 2012.^1^ For the patients who do receive a heart transplant, the median survival rate of heart transplant patients between 2002 and 2009 has been reported to be 12.5 years.^2^ The holy-grail of tissue engineering is the ability to bioengineer a total biofabricated heart, which will undoubtedly benefit heart failure patients around the world. The field of whole heart engineering has advanced significantly over the past few years, with major scientific advancements that have placed biofabricated hearts within the realm of possibilities. Advances in stem cell engineering, 3D bioprinting technology, and bioreactor development have all made the field of whole heart bioengineering a near term reality.

In this article, we provide a succinct review of the field of whole heart bioengineering, with a particular emphasis on the use of 3D bioprinting technology. We provide an overview of tissue engineering as a field and discuss different strategies that have been used to bioengineer bioartificial hearts. We provide an overview of the challenges in the field and also provide a logical and systematic process to bioprint human hearts for clinical transplantation.

## HISTORICAL PERSPECTIVE OF 3D BIOPRINTING

Physicians treating patients suffering from cardiac failure have a myriad of medical devices available to stabilize the patient and, at best delay, the progression of cardiac dysfunction. The ultimate biologic solution to cardiac failure is the replacement of the failing heart with a viable heart through allograft transplantation. More recently, advanced regenerative medicine techniques have been under development, which propose to 3D bioprint and assemble a total heart from biologic/cellular precursors. Once assembled, the heart would represent a biologic and artificial construct, and thus, the term “biofabricated” has emerged to describe these assembled biologic replacement parts. The status of 3D bioprinting technology is beyond its infancy, and an update on progress toward an implantable Total Biofabricated Heart is provided.

Bioprinting is a form of additive manufacturing. The first example of additive manufacturing was by Francoise Willeme who, in 1856, transferred photographic images to a three-dimensional physical construct that replicated the original form.^3^ With the development of the computer and plastics, additive manufacturing has rapidly emerged as a means to construct a variety of objects. The process involves computer assisted design (CAD) to provide instructions to computer assisted manufacturing (CAM) equipment that produces the object most often using a layer-by-layer additive process. The additive manufacturing of plastic parts was first described by Charles Hull in the early 1980s and has now become a major innovation in part manufacturing in the aerospace, automotive, construction, and appliance fields.^3^ The emergence of additive manufacturing in the medical field can be traced to 3D printing of surgical guides to aid physicians in the planning of complex interventions. Examples include 3D printed models of the vasculature to aid in the separation of conjoined twins and 3D printed models of the heart to assist in planning for tissue reconstruction in cardiac congenital defect patients. Additive manufacturing of implantable medical devices has been used in a growing number of clinical cases.^4^ These 3D printed implants are created using patient specific data (e.g., MRI, computed tomography scans), and the printed devices have dimensionality that matches the tissue being replaced. They are used in complex jaw, tracheal, cranial, and sternum replacements. The major innovations that have accelerated the adoption of 3D printed objects in medicine include software advances that permit rapid conversion of large image databases into a computer language recognized by 3D printing equipment and the development of relatively inexpensive 3D printers capable of rapid, high resolution plastic printing.^4^ 3D medical device printing represents a potential disruptive innovation in future medical care where devices can be produced cheaply, rapidly, and at the point of care.

The transition from 3D printing to 3D “bio”printing represents the recognition that tissue exists and functions as a three-dimensional structure with a complex arrangement of cells and extracellular matrix (ECM). Wilson and Boland are credited with the earliest work describing a method to 3D bioprint living matter into complex structures.^5^ His equipment included a HP inkjet printer where the ink cartridge was cleared of regular ink and replaced with a solution that contained a bacterial suspension. The printer was programmed to perform layer by layer deposition of this bacterial bio-ink onto a surface in a shape defined by the computer. Simultaneous with Boland's ink-jet bioprinting, investigators at the University of Arizona and Sciperio, Inc. modified a 3-axis robotic electronic printer to perform layer by layer printing of bioinks that contained mammalian cells.^6,7^ These early investigations established the ability to use CAD to CAM principles to create 3D dimensional tissues. The original 3-axis robotic bioprinter was called the Biological Architecture Tool or BAT. Early work by Jakab *et al.*^3,8^ and Mironov *et al.*^9^ also paved the way for the field of bioprinting. This earlier work was based on forming aggregates of cells to form spheroids and making use of these cell spheroids as the fundamental unit of bioprinting.^8^ In addition, this work served to demonstrate many of the important parameters for bioprinting, including cell density per spheroid and properties of the hydrogel, which impacted the bioprinting process.^8^ Almost immediately with the availability of 3D bioprinters, the question was raised: *Can we 3D Bioprint whole organs and specifically the heart?*

## DEFINITION OF TISSUE ENGINEERING

**FIG. 1.**
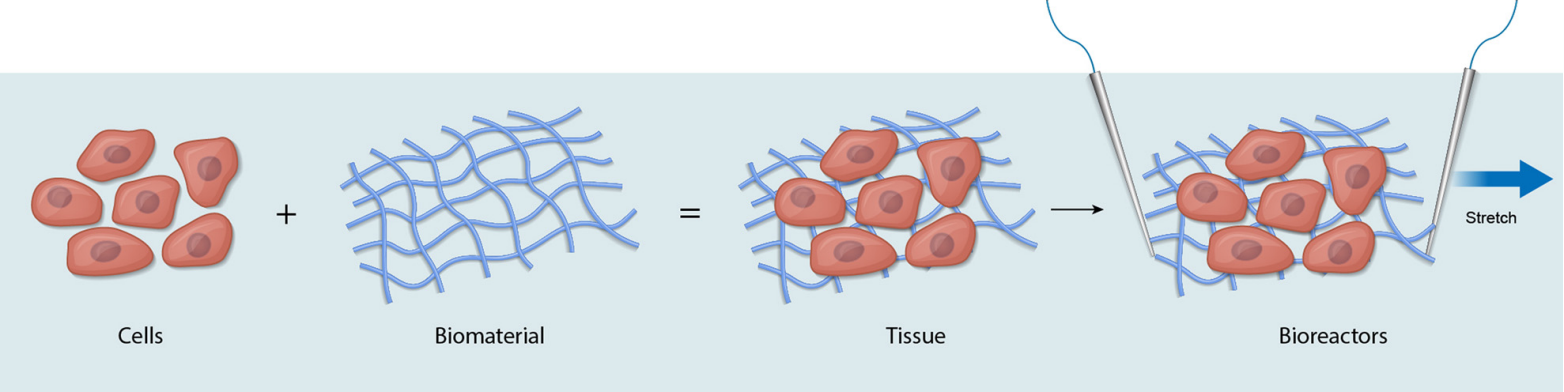
Definition of tissue engineering—the building blocks of tissue engineering are cells, biomaterials, and bioreactors. Cells are the functional elements of all tissue and organs, while biomaterials are designed to simulate the mammalian extracellular matrix and provide structural support. Bioreactors are custom devices to deliver physiological cues for 3D tissue/organ development and maturation. Electrical stimulation is delivered by parallel electrodes, while uniaxial stretch, illustrated by the single arrow, is designed to apply cyclic movement of the bioengineered tissue.

The definition of tissue engineering has been very elegantly presented in a recent publication:^10,11^ “*Tissue engineering is a multidisciplinary field bringing together experts from engineering, life sciences and medicine, utilizing the building blocks of cells, biomaterials and bioreactors for the development of 3-dimensional artificial tissue and organs which can be used to augment, repair and/or replace damaged and/or diseased tissue.*” This definition truly embodies the key elements of the field and is divided into three main components. First and foremost, tissue engineering is a *multidisciplinary field* that brings together experts from many different fields working together to solve complex problems in medicine. It is common and almost expected to witness this multidisciplinary nature in most major tissue engineering research labs and centers. Engineers, surgeons, and cell biologists are almost always seen working together in major research centers to solve complex tissue engineering problems. The second important component of the definition defines the *building blocks of tissue engineering* as *cells*, *biomaterials,* and *bioreactors* (Fig. 1). *Cells* are the functional component of any tissue and/or organ; recent advances in stem cell engineering allow the differentiation of somatic cells to almost any cell type in the human body. While cells provide the functional component of any tissue and/or organ, *biomaterials* simulate the extracellular matrix (ECM) and provide structural support during tissue fabrication and maturation. Recent advances in biomaterial design have resulted in tailormade biomaterials with tissue specific properties, mechanical properties, biocompatibility, and biomimetic activity. The third building block of tissue engineering is *bioreactors*, custom devices built to replicate the complex physiological cues within functioning tissue and organs. These cues consist of electrical impulses, mechanical stimuli, continuous fluid stresses from blood flow, and compression stresses, all of which function to support development and maturation. The third and final component of the definition outlines the potential applications of tissue engineered constructs, either for repair of replacement of damaged or diseased tissue. For examples, in cases of heart failure, 3D patches may be used to augment the functional performance of failing left ventricles, while bioengineered hearts may be used for transplantation of the damaged heart.

## THE FIELD OF CARDIAC TISSUE ENGINEERING

**FIG. 2.**
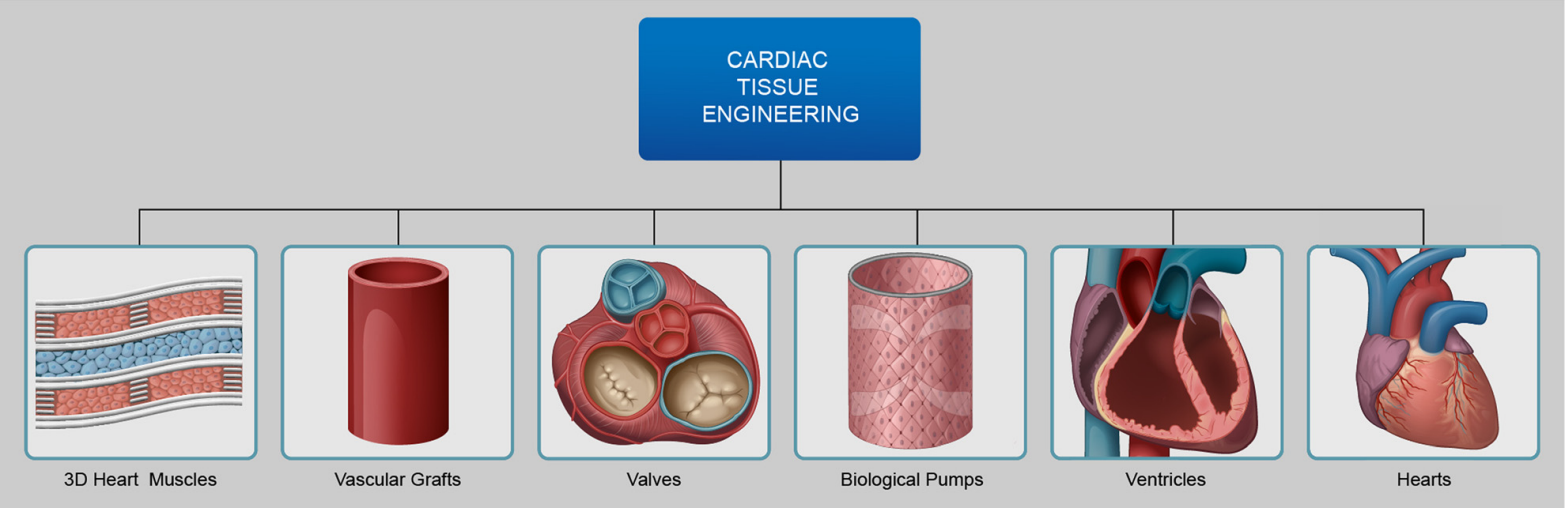
Overview of cardiac tissue engineering—the field of cardiac tissue engineering includes methods to bioengineer contractile 3D heart muscle, biological pulsating pumps, bioengineered left ventricles, bioartificial valves and vascular grafts, and biofabricated hearts. Contractile 3D heart muscle is designed to replicate the properties of mammalian heart muscle tissue and can be used as a patch to augment left ventricle pressure after myocardial infarction. Pulsating pumps are designed to generate intra-luminal pressure and can be used as biological pumps. Left ventricles can be used as a component of the heart or to replace under-performing ventricles in pediatric cases of hypoplastic left heart syndrome. Valves and vascular grafts can be used to replaced mammalian valves and blood vessels or as components of the bioengineered heart.

The field of tissue engineering is broad and encompasses all tissue and organ systems in the human body. A brief introduction to the field of cardiac tissue engineering will serve as to illustrate many of the facets of the tissue engineering as a whole. Cardiac tissue engineering is a subset of tissue engineering and targeted toward bioengineering human hearts for clinical transplantation or parts of the hearts, each with very specific target therapeutic applications (Fig. 2). The field of cardiac tissue engineering as a whole is targeted to bioengineering 3D heart muscle or cardiac patches,^12–16^ biological pumps,^17^ ventricles,^18^ valves,^19^ blood vessels,^20^ and entire bioartificial hearts,^21^ with tremendous progress being made on all fronts. Cardiac patches or 3D heart muscle are planar tissue constructs that replicate the anatomical and functional characteristics of mammalian heart muscle tissue. The potential application of 3D cardiac patches is in cases of acute myocardial infarction, where bioengineered heart muscle tissue can be used to augment contractile function. Biological pumps are tubular grafts surrounded by contractile cardiomyocytes, resulting in a hollow chambered pulsating construct, with potential applications as biological left ventricular assist devices. Bioengineered ventricles are designed to replicate anatomically and structural characteristics of mammalian left ventricles with potential applications in congenital heart surgery to treat cases of hypoplastic left heart syndrome, a condition in which neonates are born with underdeveloped left ventricles. Tissue engineered vascular grafts and valves are geared as replacement grafts in cases of coronary bypass or valve replacement surgeries. The holy grail of cardiac tissue engineering is the development of complete biofabricated hearts for clinical transplantation, the focus of the current review. The ability to bioengineer components of the heart or the entire bioartificial heart, both have applications in changing the standard of care for patients with heart disorders. Depending on the severity of the patient, a cardiac patch may be sufficient to augment lost contractile function, while in cases of chronic heart failure, a total bioartificial heart may be required.

## THE COMPLEXITY OF THE MAMMALIAN HEART

**FIG. 3.**
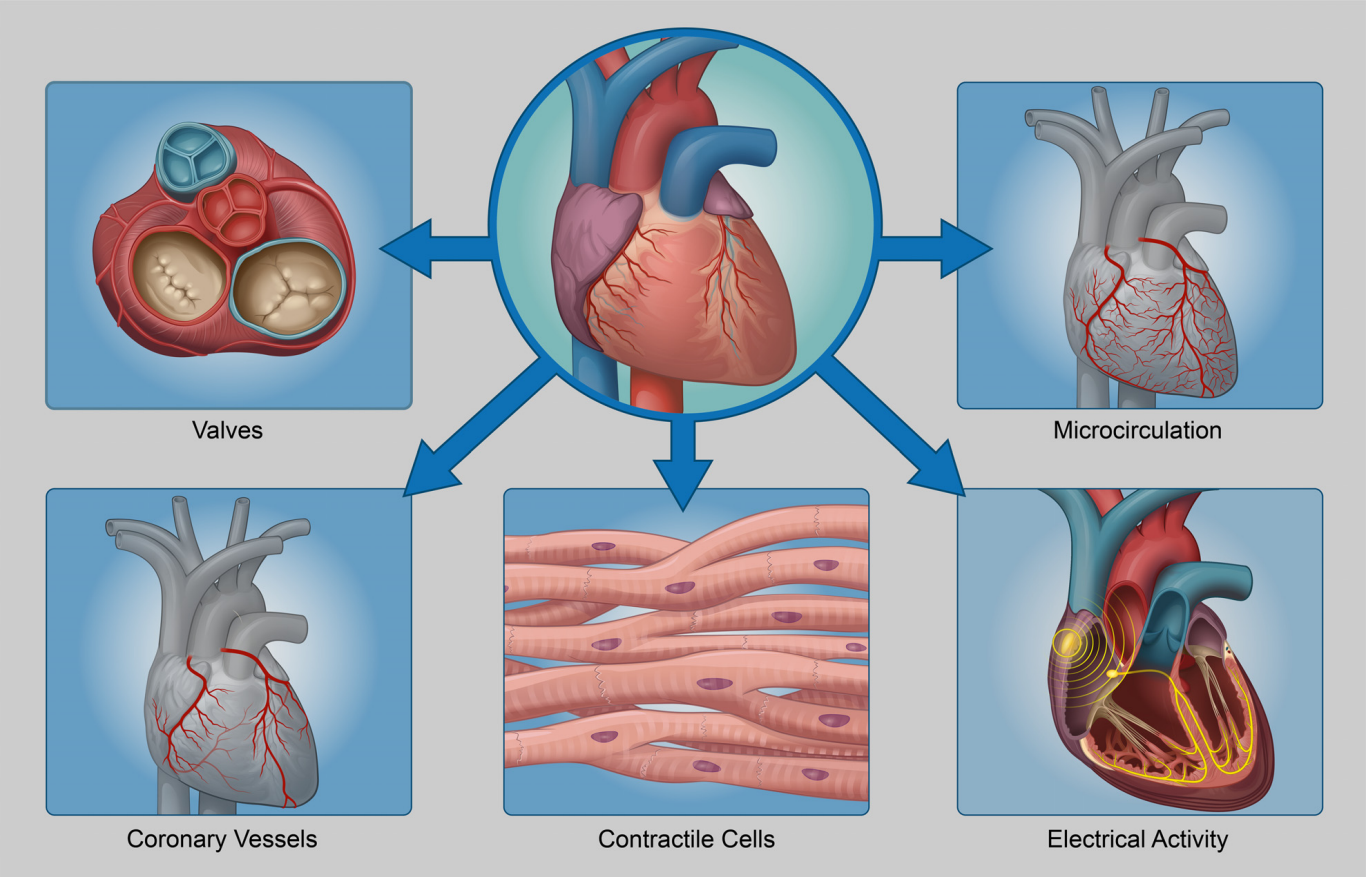
Major components of the human heart—the human heart consists of four chambers, four valves, the cardiac conduction system, contractile cardiomyocytes, and a complex vasculature. The four chambers are the left and right ventricle and aorta, while the four valves are the aortic and mitral valves and pulmonary and tricuspid valves. The cardiac conduction system consists of the SAN, AVN, bundle of His, and the Purkinje fibers. Cardiac vasculature consists of the greater vessels as well as the smaller micro-circulation. Cardiomyocytes are the cells responsible for heart muscle contraction.

The mammalian heart is a marvelous organ, one that beats an average of 70 times every minute or 2–3 billion times during the lifespan of a person, assuming an average lifespan of 75 years. From an anatomical standpoint, the mammalian heart consists of four chambers, the left and right ventricles and atrium^22,23^ (Fig. 3). Furthermore, flow of blood is regulated in the mammalian heart by four valves, two atrioventricular valves, the aortic valve, and the pulmonary valve. The mammalian heart consists of a very complex vasculature, consisting of the coronary circulation, greater vessels that permit blood flow in/out of the heart, and the microcirculation that supplies blood to the heart. An intricate balance between electrical depolarization waves and synchronized contractions of heart muscle tissue results in very fine-tuned delivery of oxygenated blood through the aorta to the entire body. The electrical system of the heart consists of the sinoatrial node (SAN), the atrioventricular node (AVN), the left and right bundle branch, and a vast network of Purkinje cells. Spontaneous depolarization waves are initiated at the SAN node, travel through the AVN, and are distributed throughout the heart via a complex network of Purkinje fibers. Depolarization of cardiomyocytes results in an increase in intracellular calcium transients, which in turn deploy a complex cascade of molecular events leading to muscle contraction. This is known as E-C coupling or coupling of electrical depolarization waves with heart muscle contraction.^24^ Spatial variations in the extracellular matrix ensure proper functioning of each component of the heart. A critical question arises in whole heart bioengineering – *how do we regulate the spatial distribution of the cells to bioengineer anatomically and functionally matched hearts?* In addition to spatial regulation of the cells, bioprinting also allows accurate placement of the biomaterials. This is where 3D bioprinting provides a powerful tool that allows us to accurately position different cell types in a very specific pattern, thereby allowing tight control over the heart bioengineering process. This applies to other tissue and organ fabrication processes, where 3D bioprinting provides a powerful tool to spatially regulate the positioning of different cell types in very specific anatomical locations.

## TISSUE ENGINEERING FOR THE HEART

There have been many publications describing the fabrication of a total bioartificial heart, almost all of which relied upon acellular scaffolds populated with either neonatal ventricular rat myocytes (NVRMs) or induced pluripotent stem (iPS) derived cardiomyocytes^21,25–30^ (Table I). In almost all cases, functional performance has been demonstrated by measuring left ventricular pressure and found to be ∼1 mm Hg. A recent publication in April 2019 showcased some preliminary success in bioprinting hearts using omental tissue as the bioink populated with iPS derived cells though functional performance was not reported.^30^ Much of the work in bioprinting hearts has served to demonstrate the initial feasibility of bioprinting hearts and has clearly moved the field from the realm of “science fiction” to “scientific reality.” This collective body of work serves to demonstrate the feasibility of bioprinting human hearts and the availability of core technologies to achieve this fate. With such a strong scientific background in place, it is only a matter of time that bioengineered human hearts will be developed for clinical transplantation. However, there remain scientific and technological challenges that need to be overcome prior to achieving this fate; as a result, it is impossible to assign a timeframe of when this will be achieved. Based on the current state of the art in whole heart bioengineering, we can safely say that human hearts will be available for clinical transplantation though we cannot assign a specific timeframe for this fate to be accomplished.

**TABLE I.** Published methods to bioengineer hearts.

Year	Senior author	Matrix	Cells	LV pressure	References
2008	Doris Taylor	Acellular rat hearts	NVRMs	1 mm Hg	27
2013	L. Yang	Acellular mouse rat hearts	iPS cells	Not reported	28
2014	I. Komuro	Acellular rat hearts	NVRMs	0.75 mm Hg	29
2014	D. Cho	Acellular bioink	Adipose cells	Not reported	25
2015	R. Birla	Acellular rat hearts	NVRMs	1 mm Hg	21
2015	R. Birla	Acellular rat hearts	NVRMs+3D patch	Not reported	26
2019	T. Dvir	Omental tissue	iPS derived cardiomyocytes	Not reported	30

## THE 3D BIOPRINTING PROCESS

**FIG. 4.**
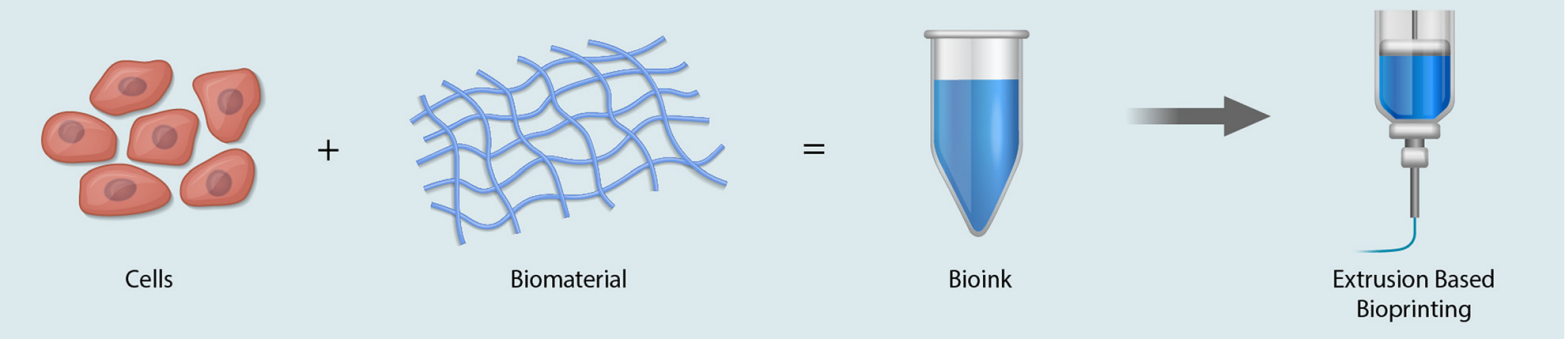
The 3D bioprinting process—isolated cells are suspended in a custom formulated bioink and loaded into a syringe. Examples of cells required to bioprint hearts include contractile cardiomyocytes, conducting pacemaker and Purkinje cells, structural fibroblast cells and vascular smooth muscle cells, and endothelial cells. Pneumatic pressure is used to extrude the cell-loaded bioink through the printing tip, and a layer by layer approach is used to build tissue and/or organs.

Extrusion based bioprinting is the most common method used in modern day bioprinters, and most commercially available bioprinters are extrusion-based systems (reviewed in Refs. 4, 31, and 32). The process for bioprinting is remarkably simple and in close resemblance to the operations of an inexpensive inkjet printer; however, the major difference is that inkjet printers deposit materials in a droplet fashion, while bioprinters deposit materials as strands. In its most simple embodiment, extrusion based bioprinting is based on isolated cells that are mixed with a bioink and loaded onto a syringe, and then, pneumatic pressure is used to move the cell loaded bioink through the syringe tip (Fig. 4). In addition to extrusion based bioprinting, there are additional modalities that include inkjet^33,34^ and laser induced forward transfer,^35,36^ which may be necessary for bioprinting at higher resolutions. The main advantage of inkjet bioprinting and laser induced forward transfer bioprinting is the high precision, higher than that obtained with extrusion based bioprinting. In the case of whole-heart bioprinting, extrusion based bioprinting will likely need to be coupled with higher resolution techniques for the placement of smaller structures, like the microvasculature and the nerves. While the selection of biomaterials used for tissue engineering is large, only a subset of these materials is suitable for applications in bioprinting. Soft hydrogels commonly used in bioprinting are fibrin, collagen, alginate, pluronic acid, agarose, and gelatin. Similar to other tissue engineering applications, the viability, purity, and concentration of the initial cell suspension being used are important. Important printing parameters include viscosity of the cell laden bioink, pneumatic pressure, printing speed, and tip diameter. High viscosity bioinks and smaller tip diameters require a higher printing pressure. The printing speed affects the diameter of the fibers, with higher speeds correlated with thinner fibers. Every bioprinting application is different and requires rigorous optimization of the bioprinting variables. Acute fine-tuning of printing parameters is required for any bioprinting application and varies significantly between tissue and organ printing applications.

## 3D BIOPRINTING HUMAN HEARTS—SCIENCE OR SCIENCE FICTION?

Bioprinting an organ as complex as the human heart was viewed as science fiction until recently. However, there have been many advancements in the field of tissue and organ fabrication, which provide a clear pathway for the bioprinting of human hearts, a field that has been transformed from science fiction to reality. This was demonstrated by a recent publication in April 2019, which showcases the ability to bioprint hearts.^30^ While this work is important in the field of human heart bioprinting, it only represents a very nominal contribution to the field. While this work showcased the ability to bioprint a 3D structure that closely resembled the human heart, there was no clear and convincing demonstration of functional performance or strong histological data that demonstrated the organization and orientation of mammalian cells. Perhaps, this work will find its place in the scientific literature as a very early demonstration of bringing together the key technological elements in the field to illustrate the feasibility to accomplishing the challenging task of bioprinting human hearts.

**FIG. 5.**
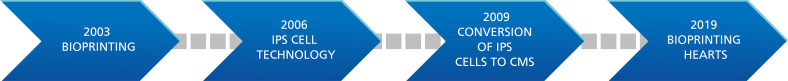
Scientific breakthroughs for 3D bioprinting human hearts.

The process to 3D bioprint human hearts is now well-established and described in detail in Roadmap for 3D Bioprinting of Human Hearts . The key elements of this process are based on several key scientific and technological advancements that have taken place during the past several years (Fig. 5). The elements of 3D bioprinting human hearts can be traced back to the first example of 3D bioprinting to create living tissue, credited to Dr. Thomas Boland in a landmark publication in 2003,^5^ Another seminal publication in 2006 revolutionized the field of tissue engineering and regenerative medicine. Adult somatic cells have been viewed as terminally differentiated cells for decades; however, in this landmark publication, it was shown that four transcription factors (October 3/4, Sox2, c-Myc, and Klf4) were sufficient to reprogram terminally differentiated skin fibroblasts to an early embryonic state, referred to as induced pluripotent stem (iPS) cells.^37^ The significance of this work can be appreciated, as the Nobel Prize for Medicine and Physiology was awarded in 2012, only six years after the initial discovery. Subsequent work in the field of stem cell engineering demonstrated the ability to convert iPS cells to practically all cell types in the human body including functional cardiomyocytes, first reported in 2009^38^ and later refined in 2013.^39^

As can be seen from the forgoing discussion, there has been rapid progress in all fronts, leading to the development of 3D bioprinted hearts. The scientific and technological elements are well established and have been proven and validated over the past several years. With such a strong and well-developed platform in place, the process to bioprint human hearts becomes a clear reality, one that moves very far away from the label of science fiction. The question is no longer—*can we 3D bioprint human hearts for clinical transplantation?* The question is now—*when will the first 3D bioprinted hearts be available for clinical transplantation?*

## ROADMAP FOR 3D BIOPRINTING OF HUMAN HEARTS

**FIG. 6.**
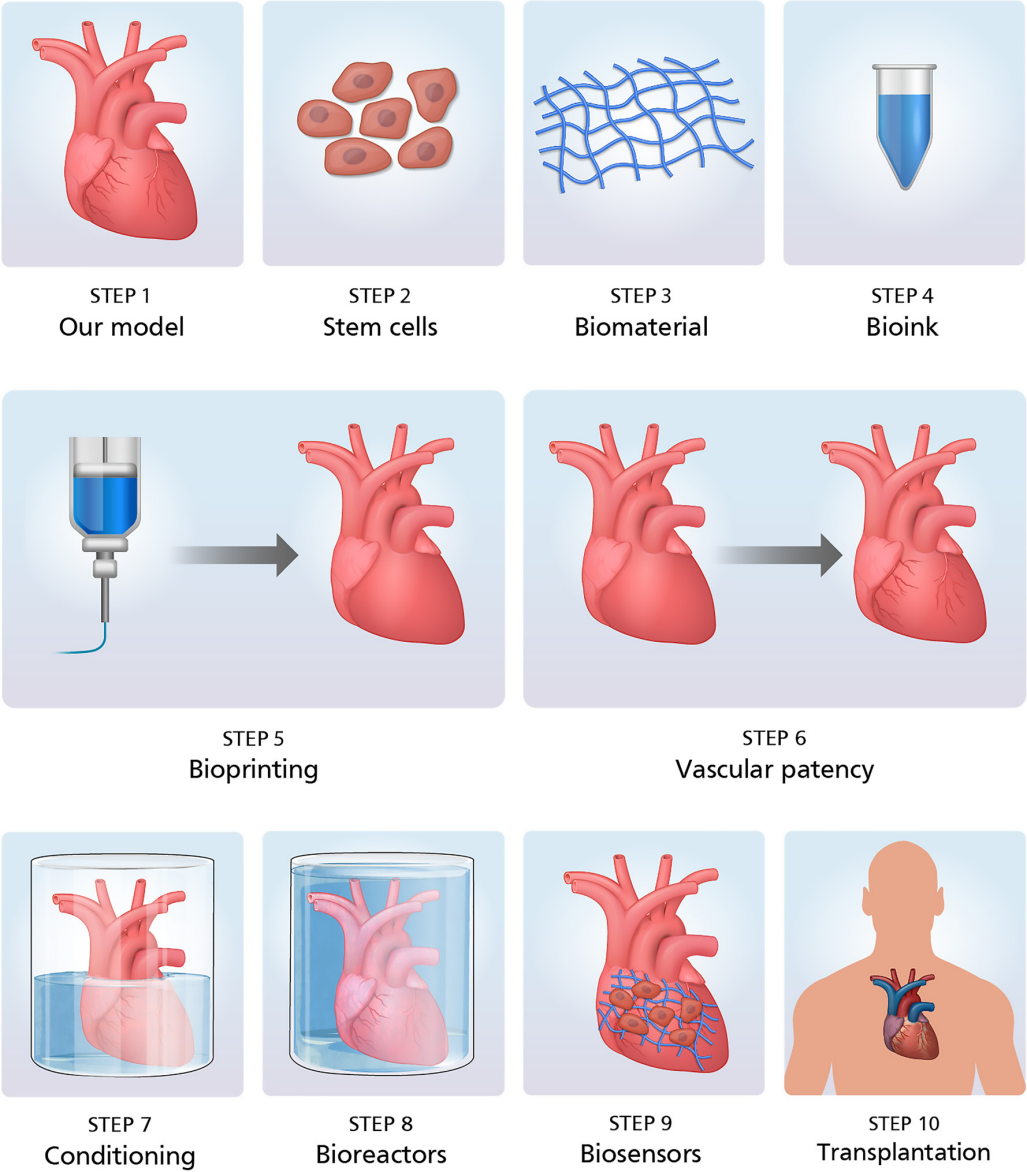
Process for bioprinting human hearts—patient MRI images are used to model the heart. Dermal fibroblasts are isolated from patient skin biopsies and converted to iPS cells and then to cardiomyocytes. Cardiomyocytes are combined with bioinks and used to bioprint patient specific human hearts. Bioprinted hearts are conditioned in bioreactors and used for transplantation.

The roadmap to bioprint human hearts is presented in Fig. 6. Patient MRIs are used to generate a complete 3D map of the human heart, one that is specific for the patient. A skin biopsy is obtained from the patient and dermal fibroblasts isolated and converted to induced pluripotent stem (iPS) cells, stem cells that have the potential to be converted to all cell types in the human body. These iPS cells are then reprogrammed to form contracting cardiomyocytes. In an ideal case, the iPS cells are also reprogrammed to form conducting pacemaker and Purkinje cells and cells of the vascular system, including smooth muscle cells, endothelial cells, and cardiac fibroblasts. The reprogrammed cells are then coupled with custom formulated bioinks, which are different for different cell types and used to bioprint patient specific human hearts. The bioinks consist of custom formulations of biomaterials, additives, growth factors, and hormones. Once printed, the hearts are cultured under static conditions for several days followed by bioreactor culture and conditioning to support heart muscle development and maturation. After bioreactor conditioning, the bioprinted hearts are ready for clinical transplantation. Custom sensors are used for real-time measurements of the cell and tissue viability, as well as the functional performance to record metrics like left ventricle pressure and electrocardiogram properties. Since the hearts were bioprinted using autologous patient cells, the bioprinted hearts are immune tolerant and the patient does not require any immune-suppression therapy.

## 3D BIOPRINTING OF THE MICROCIRCULATION

Ischemic heart disease (IHD) as a result of cardiac microvascular dysfunction is becoming increasingly recognized as a component of congestive heart failure.^40^ Moreover, recent findings indicate a higher incidence of coronary microvascular dysfunction in women vs men with an observed lower coronary flow reserve in women providing the mechanism for this gender difference coronary flow reserve.^41^ Regenerative medicine approaches to IHD and especially cell-based therapies have targeted metabolic defects that underlie the coronary flow reserve deficit.^42^ These approaches include 3D tissue constructs placed directly on the epicardial surface to address ischemic myocardium.^42–44^

A well-recognized obstacle toward the creation of a thick (i.e., greater than 500 micrometer) tissue construct and especially a biofabricated ventricular wall or the total biofabricated heart has been the inability to create a functional microcirculation to provide adequate perfusion throughout the tissue. Using fat derived vascular cells including intact microvascular fragments, investigators have successfully overcome this obstacle creating functional microvascular constructs. These pre-formed blood vessels exhibit the ability to connect to (inosculate) the recipient microcirculation, providing perfusion to thick tissue constructs.^42,45,46^ The use of adipose derived cells provides a potential autologous, point of care cell source for making these prevascularized constructs to meet the immediate needs of patients with ischemic heart disease. 3D bioprinting provides several enhancements toward vascularized tissue engineered constructs.^47^ First, the construct can be formed to fit the exact dimensions of the ischemic defect. Second, CAD/CAM based 3D Bioprinters provide a fully automated method to create the construct. This reduces operator variability and permits the assembly of the construct to be performed in a sterile environment. Finally, the development of bioinks with controllable biomechanical characteristics permits the construction of prevascularized implants with stress–strain relationships that can be printed to match the recipient tissue.

The major limitation of microvascular 3D bioprinted constructs is the dimensions of the blood vessels that can be directly assembled during the bioprinting process. The most common form of 3D bioprinting uses pen tips that deliver materials through an orifice based on a time–pressure microfluidic delivery system. The dimensions of the pen orifice, often a blunt needle, create cylinders containing the cells and binding/crosslinkable polymers. These cylinders typically have diameters not smaller than 100 micrometers, far larger than the dimensions of components of the microcirculation, namely, arterioles (20 to 80 micrometers), venules (30 to 100 micrometers), and capillaries (4 to 12 micrometers). Following 3D bioprinting of microvessel competent cells in bioinks, the cells must undergo a vasculogenic and angiogenic process to form a competent and functional microcirculation in the construct. 3D collagen gels filled with adipose derived microvascular fragments will exhibit *in vitro* 3D vasculogenesis and angiogenesis.^48^ Subsequent studies have established that adipose derived vascular cells and microvascular fragments can be 3D Bioprinted into computer-controlled shapes, and the vascular cells subsequently form microvascular structures *in vitro*. These 3D bioprinted have been implanted, and the printed and transplanted vessels form a mature and functional microcirculation.^47^

The ability to create a fully functional microvascular circulation in biofabricated tissue remains one of the most significant challenges in the field. Bioprinting technology has addressed this challenge in a unique way, with the ability to create a functional microvascular. Other technologies like whole-organ decellularization have tackled this problem differently, making use of the existing vasculature for organ perfusion. However, neither of these two technologies have matured to the point of producing a fully functional microvascular, one that resembles that of mammalian tissue. While this has not been achieved, with recent advances in the field of tissue engineering, it is only a matter of time when this problem has been solved.

## CONSTRUCTION OF CORONARY MACROVASCULAR STRUCTURES

Atherosclerosis of the coronary circulation remains the major cause of cardiac failure. The search for a biologic coronary artery bypass graft remains a focus of numerous laboratories with the goal of achieving function equal to native vessel bypass grafts. The Achilles heel of these alternative CABG conduits has been early thrombosis due to the lack of a mature endothelium on the luminal surface. The process termed *in vitro* endothelialization has been used to create autologous endothelial cell linings on synthetic grafts;^49^ however, the process is time consuming, requiring 6 to 8 weeks of maturation before the vessels are ready for implantation. Point of care, autologous endothelial cell seeding of grafts has been evaluated extensively in pre-clinical models and several clinical trials are ongoing testing this approach to a more biologic alternative arterial bypass graft.^50^ The original approach using 3D bioprinting to create small diameter vascular conduits used a spheroid approach to create the conduits followed by an extensive maturation period to permit maturation of the biologic conduit.^8^ A more recent 3D bioprinting approach to large vessel conduits involves the use of a modified print head that directly extrudes tubes of varying sizes.^51^ These tubes can be of controlled diameter and length; however, the time required for maturation of these vessels currently does not permit immediate implantation following printing.

An intriguing aspect of computer assisted design when planning the construction of a bioprinted heart is the ability to correct inherent deficiencies in the human heart. For example, the coronary vascular system in the human heart has no redundancy features, and without collateral circulation, occlusions of coronary arteries result in acute myocardial infarction and its sequelae. When designing a bioprinted heart of the future, the possibility arises to build in redundancies and provide multiple conduits to perfuse cardiac muscle.

## 3D BIOPRINTING FOR THE CARDIAC CONDUCTION SYSTEM

Another critical aspect will be the ability to reengineer the cardiac conduction system, which is a very delicate and intricate system designed to distribute synchronized depolarization waves throughout the heart. The SAN consists of a cluster of specialized pacemaker cells located at the junction of the superior vena cava with the right atrium and is responsible for generating spontaneous pacemaker activity of the heart.^52–54^ Electrical impulses generated at the SAN node travel through the AVN, through the bundle of this, and the throughout ventricular tissue via specialized Purkinje fibers. While the cardiac conduction system is complex, the SAN can be viewed as the point of initiation of electrical activity, while the Purkinje fibers are largely responsible for transmitting the electrical activity through ventricular tissue leading to heart muscle contraction.

Atrial and ventricular arrhythmias remain a significant clinical problem in patients with heart failure. While there are some effective pharmacologic therapies as well as invasive ablation techniques, it is often difficult to control these arrhythmias. Cellular and regenerative medicine based approaches for the treatment of arrhythmias remain in their infancy with some evidence of cell based restoration of normal conduction with potential restoration of sinus rhythm.^55^ The use of 3D bioprinting technology will most likely have little impact on the direct treatment of arrhythmias with the possible exception of being able to implant new spontaneously depolarizing cells. On the other hand, 3D microphysiologic models of cardiac tissue that includes printed conduction systems or cells derived from patients with genetic predisposition to arrhythmias provide important *in vitro* models supporting the development of effective anti-arrhythmia drugs.

The future bioprinting of the Total Biofabricated Heart will undoubtedly benefit from emerging 3D maps of the human heart conductive system. The source of cells for the bioinks that will be used to print this conductive system is not established but will undoubtedly be derived from a modified muscle cell population. While the exact mechanism of bioengineering the cardiac conduction system has not been worked out, the idea is to reprogram iPS to early cardiac progenitor cells and then to both pacemaker and Purkinje cells. The programmed cells can then be used to build artificial AVNs, SANs, and Purkinje fibers, critical components of the cardiac conduction system and Biofabricated Hearts.

There are prevalent cardiac rhythm diseases that may benefit from regeneration of human Purkinje and pacemaker cells. For example, sick sinus syndrome (SSS) shows that the heart's natural electrical pacemaker, the SAN, is not working properly.^56–59^ In SSS, the heart rate can alternate between slow (bradycardia) and fast (tachycardia). Treatment for SSS is usually an artificial pacemaker, along with medication. In premature contractions, extra, early, or “skipped” beats are the most common cause of irregular heart rhythms. Heart block occurs when electrical signals from the upper chambers of the heart (atria) cannot travel to the lower chambers (ventricles), and heart block happens. The heart then beats too slowly, decreasing the amount of oxygen that gets to the body and brain. Long QT Syndrome (LQTS) is a disorder of the electrical system that can be inherited and at risk for ventricular fibrillation (VF), the most dangerous heart rhythm that causes sudden death. The idea of treating rhythm diseases will be of great clinical significance, and replacement therapy with conduction cells may be an important step in restoring lost myocardial functionality.

## 3D BIOPRINTING FOR HEART VALVES

A number of valve pathologies result in cardiac failure. Valve replacement therapy remains highly successful prolonging the life of patients in a selected number of patients. With the advent of percutaneous valve replacement, the opportunity to treat a wider range of patients including patients who are not good surgical candidates is changing the treatment timing and valve repair/replacement strategies. Some investigators predict a time when routine non-surgical valve replacement/repair will be a reality. With the development of rapid, highly validated CAD/CAM processes and new polymers for 3D printing, we are on course to potentially see 3D printed valves enter the clinical arena. This will be truly a disruptive innovation where the valves can be made cheaply and at the time of implantation based on anatomic models provided by the patient's own cardiac images. The regulatory hurdles will be daunting, and it is expected that 3D printed valves will first enter human use in those countries where finances present the major barrier to the use of currently Food and Drug Administration (FDA) approved replacement valve and annuloplasty ring type devices. The future will undoubtedly see the use of 3D printed composite valves with percutaneous delivery systems.

Progress is being made using 3D bioprinting technologies to design and print valves composed of mammalian cells.^60,61^ These investigators have utilized CAD/CAM methods to produce anatomically correct valve structures composed of valve derived cells and bioink biomaterials that provide initial strength to the construct. Again, significant work remains to establish the conditions that permit correct maturation of the valves to achieve biological and biomechanical functions that replicate the human valve. The development of cellular coatings to achieve an antithrombogenic lining on all surfaces will benefit from ongoing studies creating endothelial cell linings on vascular conduits.^50^

For the final assembly of the total biofabricated heart, it seems likely that the valves will be bioprinted separately from the specific chambers of the heart, and robotic placement of the valves into their anatomic positions will occur near the completion of the heart. Again, we are considering the best design for a 3D bioprinted valve using the human heart valve anatomy as a starting point.

## 3D BIOPRINTING FOR CARDIAC MUSCLE

The fifth component of the total biofabricated heart is the cardiac muscle with its central role to create contraction and filling of the chambers of the heart. The search for an autologous source of cardiomyocytes remains under intense investigation and will certainly benefit from new understanding of cell differentiation. Induced pluripotent stem cell technology holds great promise to provide autologous cardiomyocytes derived from a patient's own progenitor cells.^55,62–64^ 3D microphysiologic systems are under development utilizing stem cell derived cardiomyocytes derived from patients with a variety of cardiomyopathies.^65^ 3D bioprinting of cardiomyocyte tissue constructs or mixed populations of cells including cardiomyocytes and vascular cells holds great promise for drug discovery.^66^

During assembly of the total biofabricated heart, the 3D bioprinting process will most likely integrate the placement of cardiomyocytes with specific spatial orientation, the microcirculation, and electrical conductivity elements. It is anticipated that this cardiac tissue construct will also be integrated with a macrovascular conduit that provides both arterial perfusion and venous return. We have assembled a microvascular and macrovascular system into a structure we define as a dynamic *in vitro* perfusion (DIP) chamber.^67^ This will form the basis for a surgically implantable vascular system that will ultimately be assembled using 3D bioprinting to include cardiomyocytes.

The clinical targets for this next generation of 3D Bioprinted cardiac tissue will include pediatric patients and especially those patients with congenital defects resulting in insufficient tissue to permit reconstruction of the heart. A significant advantage of 3D Bioprinted tissue constructs will be the ability of these constructs to grow with the child. Clearly, it remains unknown how these complex 3D bioprinted and implanted cardiac tissues will grow and adapt to the physiologic signals that regulate normal organ development and maturation. Studies of tissue engineered blood vessels implanted as pulmonary artery replacements indicate that the vessels mature and continue to grow with the patients.^68^ Future studies will be necessary to establish how all the components of a total biofabricated heart develop and function following implantation.

Any new technology must be fully validated prior to acceptance, and 3D printing and 3D bioprinting for medical applications will be no exception. The concept of printing a model of the heart to assist in evaluation of disease and to help direct proper intervention has become reality, however; this new capability brings new questions regarding how quickly this should enter clinical practice.^69,70^ All components of the process of 3D printing will be scrutinized from image acquisition to the accuracy of the final print. The cost of this entire process will be assessed. 3D bioprinting will require even greater scrutiny as the final product must provide efficacy and durability that matches the tissue being replaced. The opportunity arises from all these challenges that we may, in the future, be able to program a 3D bioprinter to assemble a part of the heart or even the total biofabricated heart based on designs that overcome the deficiencies in the human heart, which lead to cardiac failure.

## CHALLENGES IN 3D BIOPRINTING OF HUMAN HEARTS

We have presented a clear and logical pathway to bioprint human hearts as well as the key scientific and technological challenges that have moved the field to this point. Much progress has been made during the past several years, and it is now clear that 3D bioprinted hearts for clinical transplantation are a near term reality. However, as with any scientific endeavor, the field of 3D bioprinting human heart is not without its challenges. The single most important challenge that needs to be overcome in the field, and one that in general staggers the field of cardiac stem cell therapy, is the immaturity of reprogrammed cardiomyocytes. Conversion of iPS cells to cardiomyocytes is now standard and reproducible, the differentiated cells resemble an embryonic phenotype, and driving these cells to an adult phenotype remains a critical challenge in the field of cardiac stem cell therapy. A recent publication addressed this challenge and showcased that coupled electromechanical stimulation of cardiomyocytes reprogrammed from iPS cells showed markers of adult phenotype, including the presence of well-organized endoplasmic reticulum and sarcoplasmic reticulum.^71^ While this work addresses a clear need in the field of cardiac stem cell therapy and 3D bioprinting of human hearts, it is yet to be reproduced in other labs, mainly due to the use of specialized bioreactors for electromechanical stimulation used in the published study. Once reproduced by independent research labs, coupled with the availability of commercial bioreactors for electromechanical stimulation, the availability of mature cardiomyocytes will provide a clear pathway to 3D bioprint human hearts for clinical transplantation. In addition to the maturation of iPS derived cardiomyocytes, there remain many other challenges. Conversion of iPS cells to cardiomyocytes is a very specialized skill and requires trained technical staff. Maintaining iPS cells in a pluripotent stage remains challenging. There is a high cost associated with the production of iPS derived cardiomyocytes, due to the cost of associated reagents and also due to the required training level of research staff. Furthermore, there are currently challenges in producing a very large number of iPS derived cardiomyocytes required to bioprint human hearts.

Recent advances in the field of 3D bioprinting have provided a clear pathway for the future, demonstrating the tremendous potential bioprinting has in developing functional organs for clinical transplantation. While there remain challenges in the field from a scientific and technological viewpoint, there are also challenges related to regulatory factors. Many of the challenges associated with regulatory issues in 3D bioprinting are the same as the field of tissue engineering in general and are somewhat vague in their scope. This has been attributed to the lack of large-scale commercial success in the field of tissue engineering broadly and 3D bioprinting more specifically. As the field matures to deliver commercial successes, there will be parallel advances in the regulatory process with more clarity in scope.
